# Evaluation of early apical release with bipolar Collins knife versus Thulium-Yag laser enucleation of large-sized prostate. A randomized study

**DOI:** 10.1080/20905998.2024.2321737

**Published:** 2024-02-23

**Authors:** Ahmed Gamal Eldin, Mohammed Abdallah, Ahmed Fouad, Mohammed Omar

**Affiliations:** Faculty of Medicine, Urology Department, Menoufia University, Shibin el Kom, Egypt

**Keywords:** BipolEP, ThuLEP, AEEP, large, prostate

## Abstract

**Introduction and objectives:**

Anatomical endoscopic enucleation of the prostate (AEEP) provides durable management for patients with lower urinary tract symptoms (LUTS) secondary to large-sized prostate over other surgical modalities. We aimed to assess the early outcomes of Collins knife-assisted bipolar enucleation (BipolEP) versus Thulium-Yag enucleation (ThuLEP) in a group of patients with LUTS secondary to a prostate larger than 80 grams.

**Methods:**

We included patients with benign prostatic hyperplasia (BPH) having a prostate volume > 80 grams, international prostate symptom score (IPSS) >7, urine flow (Q-max) <15, and post-void residual (PVR)>150 ml. We excluded those with a history of previous prostatic surgery, stone, or neurogenic bladder. Bipolar enucleation with early apical release was performed using Collins knife at an 80/100-watt setting (Lamidey Noury), while ThuLEP was conducted using 550- micron fiber and 40/15-watt energy (Lisa Laser). Patients were evaluated before then 2 weeks and 3, 6,12 months postoperatively for changes in IPSS, Q- max, PVR, and the incidence of stress incontinence.

**Result:**

One hundred and twenty patients were equally randomized with a mean prostate size of 104 ± 25 gram. The mean IPSS score was 25 ± 6, Qmax 7.6 ± 1.3 mL/S, and PVR 225 ± 39. There was no significant difference regarding enucleation time, morcellation time, and enucleated tissue volume. Irrigation volume and post-operative hemoglobin drop were significantly lower in the bipolar group (*p* = 0.008, *p* = 0.0002), respectively. At the third-month follow-up, IPSS, Q-max, and PVR were comparable across both groups, with stress incontinence at 3.3% in the bipolar group versus 1.6% in the thulium group, showing an insignificant difference (*p* = 0.5).”

**Conclusion:**

Both BipolEP and ThuLEP, with early apical release, provide a safe and effective management of large-size prostate resulting in significant decrease in post-operative stress incontinence incidence during early follow-up. Intraoperative irrigation saline volume, and post-operative hemoglobin drop favored the bipolar group.

## Introduction

Although transurethral resection of the prostate (TURP) has been the primary treatment modality for lower urinary tract symptoms (LUTS) secondary to benign prostatic hyperplasia (BPH) (LUTS/BPH); now is the time for a change. ^*(1)*^ Anatomical endoscopic enucleation of the prostate (AEEP) is considered the alternative; especially for patients with prostates exceeding 80 grams, to improve surgical outcomes, reduce the need for re-treatment^,^^*(2)*^ with equal efficacy and durability compared to open prostatectomy. ^*(3)*^

Hiraoka and Akimoto introduced bipolar enucleation without morcellation [1] 10 years before Gilling [2] used Holmium enucleation of prostate (HoLEP). Encouraged by their success, the New Zealand group [3] published the first randomized trial comparing HoLEP versus bipolar enucleation of the prostate (BipolEP) with similar outcomes. In 2010, the time came for Herrmann [4] to present their technique for Thulium enucleation of prostate (ThuLEP). Large-size prostate is the primary subcategory of BPH patients who benefit the most from enucleation, and the durability of TURP procedures may be progressively reduced depending on the prostate size and patient longevity [5]. Early apical release is the cornerstone in the perspective of most endourologists for reducing the risk of stress incontinence after enucleation.

Bipolar enucleation using the new bipolar Collins knife technique may have the advantage of being a less expensive technology, providing early apical release and reducing the risk of blood loss during AEEP in these large prostates. Accordingly, Thulium-Yag enucleation uses a continuous wave (CW) operation that allows for precise cutting and coagulation, with the ability to produce minimal thermal damage to surrounding tissue. In this study, we aimed to compare the safety and effectiveness of Bipolar enucleation of the prostate with the new Collins knife technique versus the Thulium-Yag laser f or the treatment of benign prostatic hyperplasia in patients with more than 80-gram prostate.

### Methods

Following Institutional review board approval, we included BPH patients with prostate volume >80 ml, IPSS score > 7, urine flow (Q-max) <15, and post-void residual (PVR)>150 ml, who were admitted to the Menoufia Department of Urology between November 2018 and April 2022 after obtaining successful consent. Randomization was performed with a 1:1 allocation using a computerized random number table to assign patients to BipolEP using the new Collins knife technique (group A) or ThuLEP (group B).

Sample size calculation revealed 52 patients per group using the clinics calc sample size calculator [6], setting the type-1 error (α) at 0.05 and power at 80%. All patients underwent thorough preoperative examination and laboratory testing. Preoperative data, intraoperative variables and postoperative outcomes were recorded and followed up for 3, 6, and 12 months. The primary endpoint of our study was the mean reduction of hemoglobin after surgery and secondary endpoints included operative time, irrigation volume, time to control bleeders, enucleated tissue, hospital stay and stress incontinence.

#### Operative procedure

**Figure 1.**
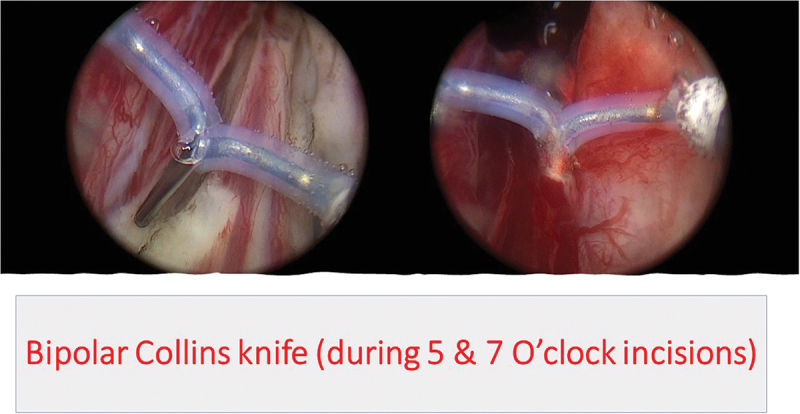
Bipolar knife (during 5&7 O’click incisions)

**Figure 2.**
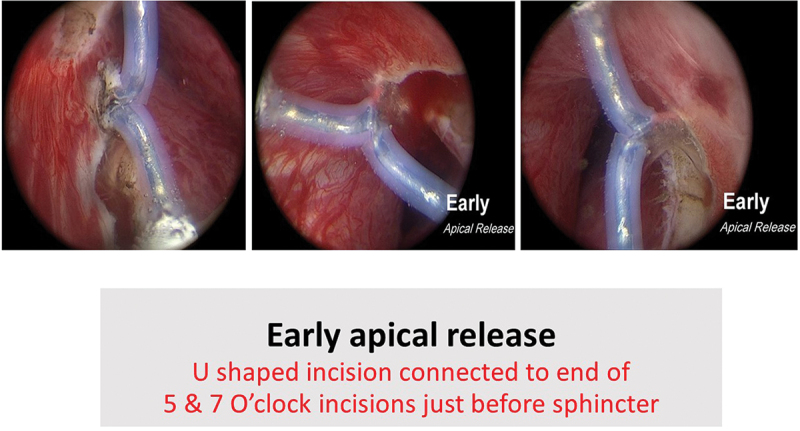
Early apical release

**Figure 3.**
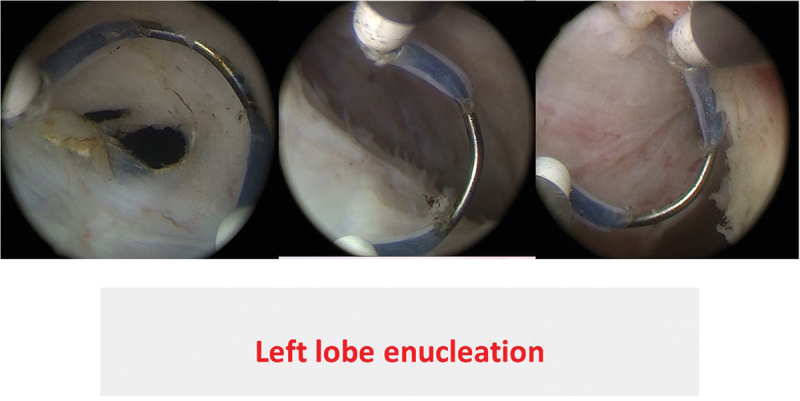
Left lobe enucleation

All procedures were performed by an experienced urologist (M.O) with AEEP experience in more than 400 cases. In group A, the Collins knife (Lamidey Noury Medical ® Paris, Verrières le Buisson, France) was used to incise the prostatic mucosa at the 5 and 7 O’clock positions (Figure 1.) from bladder neck down to above Verumontanum in all bi-lobar cases. For tri-lobar prostates, incisions were joined together before the Verumontanum, followed by an inverted U-shaped incision (Figure 2.) connected to the end of 5 & 7 O’clock incisions just before the external urethra sphincter, to achieve early apical mucosal release. This was done using a cutting/coagulation setting of 80/100 Watts. Subsequently, a specific enucleation electrode, PlasmaLEP™(Lamidey Noury Medical ® Paris, Verrières le Buisson, France), with a cutting/coagulation setting of 100/120 Watts using the electrosurgical unit OPTIMA™(Lamidey Noury Medical ® Paris, Verrières le Buisson, France) was used for enucleation of the median and lateral lobes (Figure 3.):

In group B, the technique was typically the same as mentioned in group A, but Thulium-Yag laser was used instead. This was followed by assisted mechanical enucleation by the tip of the resectoscope and laser for incision and hemostasis. A cutting/coagulation setting of 40/10 watt of a RevoLix™ (LISA Laser Products OHG, Katlenburg-Lindau, Germany) delivered via 550 μm end firing Percu Fib (LISA Laser Products OHG, Katlenburg-Lindau, Germany).

After complete separation of prostatic lobes into the bladder and brief hemostasis, morcellation was performed using Drillcut-X II (KARL-STORZ® Tuttlingen, Baden-Wurttemberg, Germany) 2500/rpm then the morcellator speed was reduced to 1500 rpm when smaller-sized lobes turned away from the morcellator. A 20-F silicone catheter was inserted, with intermittent irrigation when urine turned red. The catheter would be removed on the first day of surgery, and the patient would be discharged after a micturition trial.

### Statistical analysis

Results were tabulated and statistically assessed using SPSS 25 (SPSS Inc., Chicago, IL, USA) and Microsoft Excel 2017 on a personal computer. Descriptive data, such as percentage (%), mean, and standard deviation, were used for statistical evaluation. Analysis methods included the t-test, paired t-test, Mann-Whitney test, and chi-squared (x2) test. A P-value less than 0.05 was considered statistically significant.”

## Results

**Figure 4.**
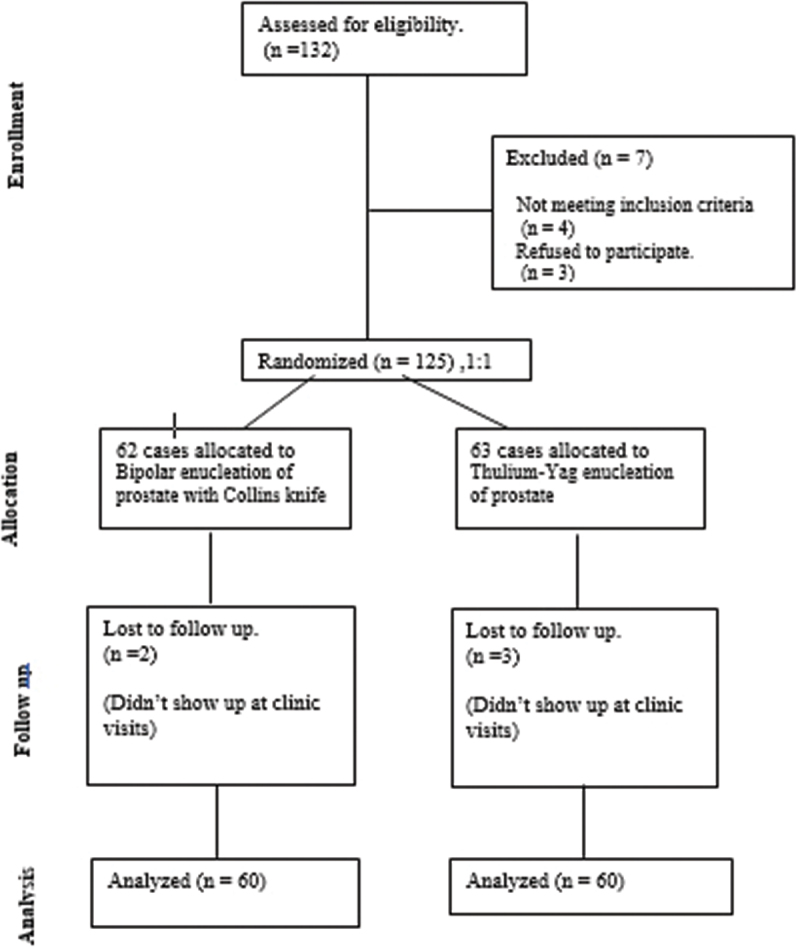
Consolidated standards of reporting trials (CONSORT) flowchart of study cases.

We successfully consented, randomized, and allocated 125 patients into the BipolEP (group A) and ThuLEP (group B) groups. Figure 4 The mean patient’s age and prostate size were 65 ± 7 years and 104 ± 26 grams, with comparable preoperative characteristics (Table 1). Bipolar and laser prostate enucleation did not substantially differ in terms of operational outcomes, such as enucleation time/min, morcellation time/min, enucleated tissue volume (ml), and hospital stay (*p* > 0.05). (Table 2). However, the BipolEP groups showed shorter time to control significant bleeder (5 vs. 22 sec, *p* = 0.0001), less intra-operative irrigation utilization (50.5 vs. 54.6 liters in ThuLEP, *p* = 0.008), less postoperative change in hemoglobin (13.6 vs. 12.9 in ThuLEP, *p* = 0.03), and less hemoglobin drop (0.58 vs.1.14 in ThuLEP, *p* = 0.0002) with significant differences.

**Table 1.** Preoperative characteristics.

Variable	Bipolar enucleation (Group A) (*N* = 60)	Laser enucleation of (Group B) (*N* = 60)	*p value*
Age (years) (Mean± SD)	65.25 ± 6.4	64.6 ± 6.7	0.65
Prostate volume (ml) (Mean± SD) (Median, IQR)	105.04 ± 27.77 99(88–112)	103.1 ± 24.55 94(86–114)	0.487
PSA (ng/ml) (Mean± SD)	3.3 ± 4.1	3.7 ± 3.4	0.211
IPSS (Mean± SD)	25.39 ± 5.74	24.91 ± 6.38	0.791
Q max (Mean± SD)	7.52 ± 1.20	7.86 ± 1.73	0.425
PVR (Mean± SD)	236.12 ± 54.12	212.79 ± 34.38	0.315
Hemoglobin (Mean± SD)	14.1 ± 0.85	13.85 ± 1.06	0.906
Diabetes mellitus	3(5%)	5(8%)	0.3
Hypertension	6(10%)	7(12%)	0.7

PSA prostate specific antigen, IPSS international prostate symptom score, Q max Maximum flow rate, PVR post-void residual volume.

**Table 2.** Peri- operative outcomes.

Variable	(Group A)	(Group B)	*p value*
Enucleation time/min (Mean± SD)	77.87 ± 27.96	76.36 ± 16.41	0.865
Morcellation time/min (Mean± SD)	16.47 ± 2.78	15.91 ± 3.41	0.350
Enucleated tissue (gram) (Mean± SD)	71.22 ± 16.97	70.05 ± 14.19	0.727
Irrigation volume/liter (Mean± SD)	50.55 ± 6.63	54.61 ± 8.18	0.008
Post-operative hemoglobin (Mean± SD)	13.59 ± 0.4	12.94 ± 1.04	0.0318
Hemoglobin drop (Mean± SD)	0.58	1.14	0.0002
Time to control significant bleeder/sec (Mean± SD	5 ± 2	22 ± 12	0.0001
Hospital stay (days) (Mean± SD)	1.1 ± 1	1.2 ± 1.5	0.788

For all included patients, the mean day of discharge for the bipolar and laser groups was (1.1 vs 1.2 days), (*p* = 0.7), and the catheter was removed before discharge. Early postoperative complications were comparable in both groups, with only two cases requiring blood transfusion in the ThuLEP group (*p* = 0.09), while one case required readmission after one week in the BipolEP group due to fever and urinary tract infection (*p* = 0.2). The rate of stress urinary incontinence was 8 & and 7% in BipolEP and ThuLEP, respectively (*p* = 0.7) during the 2^nd^ week postoperative visit (Table 3).

**Table 3.** Postoperative complications (2 weeks).

Complication	(Group A)	(Group B)	P value
Blood transfusions	0	2(3.6%)	0.09
Stress incontinence (2^nd^ week postoperatively)	5 (8.3%)	4(6.6%)	0.7
Re-admission	1(1.6%)	0	0.2
Bladder injury	0	0	1

Regarding the IPSS (2.50 ± 0.71 vs. 2.30 ± 0.35), PVR (43.13 ± 17.92 vs. 33.75 ± 15.53), Q max (19.38 ± 1.41 vs. 21.00 ± 2.20), and stress incontinence (3.3% vs. 1.6%) all groups showed equivalent progress at follow-up with an insignificant *p* value (p > 0.05), (Table 4).

**Table 4.** Follow-up variables.

Variable (3rd,6^th^, 12^th^ follow-up)	(Group A)	(Group B)	P value
Stress incontinence			
3^rd^ month	3.3%	1.6%	0.5
6^th^ month	1.6%	1.6%	1
12^th^ month	1.6%	1.6%	1
IPSS (Mean± SD)			
3^rd^ month	2.50 ± 0.71	2.30 ± 0.35	0.733
6^th^ month	3.94 ± 0.94	4.10	0.65
12^th^ month	4.67 ± 1	4.95 ± 1.2	0.32
Q max (Mean± SD)			
3^rd^ month	19.38 ± 1.41	21.00 ± 2.20	0.880
6^th^ month	18.8 ± 2.9	20.3 ± 3.1	0.44
12^th^ month	21.6 ± 2.7	20.2 ± 2.2	0.65
PVR (Mean± SD)			
3^rd^ month	43.13 ± 17.92	33.75 ± 15.53	0.078
6^th^ month	39.55 ± 21.68	32.55 ± 14.58	0.09
12^th^ month	40.61 ± 23.64	33.55 ± 20.66	0.09

HB: Hemoglobin, IPSS: International prostatism symptom score.

Q max: Maximum urinary flow rate, PVR: Post-void residual urine.

## Discussion

Benign prostatic hyperplasia (BPH) is one of the most common diseases affecting middle-aged and older men frequently [7], resulting in significant lower urinary tract symptoms (LUTS). People who experience LUTS feel uncomfortable, and their quality of life (QoL) is often significantly reduced. Minimally invasive prostatectomy will be the leading solution in more than 20% of moderate to severe LUTS cases after the age of 50 [8].

Transurethral resection of the prostate (TURP) has been one of the most performed endoscopic prostatectomies since 1949 [9]. Using a bipolar electrosurgical unit allows saline (NaCl 0.9%) irrigation and overcomes the major disadvantage of monopolar TURP.

In the last 25 years, laser beams have illuminated the pathway for endoscopic enucleation of the prostate, imitating the open enucleation principle without its comorbidities and providing durable, long-standing outcomes utilizing Holmium Yag, Thulium-Yag, Greenlight, or even Thulium fiber laser. The only obstacle was the steep learning curve that could be exceeded in the last seven years with the widespread use of different techniques worldwide. Accordingly, the bipolar shine did not fade out as the bipolar enucleation was ready to join the same endoscopic convoy.

Many clinical trials have investigated the outcomes of anatomical endoscopic enucleation of the prostate (AEEP) against open prostatectomy or TURP. The short- and long-term effectiveness of AEEP is more evident in cases with large prostates where the superiority of enucleation over TURP cannot be denied.

In this study, we prospectively compared the safety and effectiveness of BipolEP versus ThuLEP for the treatment of patients with prostates larger than 80 grams. There were no statistically significant differences between the studied groups in all demographic variables. The choice of larger prostate size would be of more excellent value to show the intra and postoperative outcomes of both bipolar or Thulium energy and the procedure. Many previous comparative studies like Liu [10] et al., and Song et al. [11] had a mean prostate size of around 65 grams, which may not uncover the difference between both energy sources.

It is well known that bipolar energy has the most significant depth of tissue penetration in comparison to Thulium-Yag [12] laser. In vitro studies showed, a 180 µm coagulation depth for a 30-watt Thulium Yag setting in comparison to 280 µm for a bipolar electrode with a significant difference [13]. Raising the Thulium-Yag setting to more than 30 watts is inadequate for coagulation, while most experienced surgeons may even use a setting of less than 30 watts for coagulation.

Unlike HoLEP, ThuLEP and BipolEP employ incision of the prostatic tissue down to the surgical capsule followed by blunt enucleation with the sheath of the resectoscope and point cauterization of significant bleeders. Such more mechanical enucleation may favor a large caliber energy source to deliver a non-penetrating hemostatic wave to stop the capsular perforator without violating the capsule.

Such a significant difference with utilizing a large caliber electrode (PlasmaLEP™) compared to the slim tip 550 Micron laser tip would lead to a large coagulation zone width and depth [14] and heat damage zone [15], that explain the more hemostatic superiority results for the bipolar over Thulium-Yag laser in our study.

Again Liu [11] et al., and Song et al. [12] reported less hemoglobin drop for the ThuLEP arm, which contradicts our results that confirm the superiority of BipolEP for providing less hemoglobin drop and a decrease in the intra-operative volume of used saline. Perhaps such a difference could be related to the technique of performing enucleation in the bipolar group that was not mentioned in Liu’s study, which may be a regular TURP, especially when performing a tangerine rather than the standard ThuLEP technique.

Another central point in these two studies is the lack of a morcellator, which may result in the need for more resection of the partially separated lobe, which may lead to another episode of bleeding that may be avoided by utilizing a high-watt setting of Thulium-Yag laser for vaporization of the partially enucleated adenoma. This could explain why both Liu [11] et al., and Song et al. [12]; were unable to account for the volume of resected tissue.”

The bipolar group have significant lower mean intraoperative irrigation (4 liters less than the thulium) and shorter time to control bleeders (5 vs 22 sec); although the similar intraoperative time, that could be explained by the more use of pressurized irrigation during bleeders cauterization which increase the amount of utilized irrigation.

A mean 75-gram prostate indicates more than 20 bleeders, of which 8 are significant ones [16]. Our mean prostate size was over 100 grams for both groups, posing a challenge for enucleation surgery where the diameter of prostatic perforator vessels or the number of significant bleeders per case is strongly related to the prostate size. Also, we observed that BipolEP had a shorter time to control significant bleeders, reaching just 5 seconds, compared to ThuLEP, which took 22 seconds, reflecting greater hemostatic efficiency in demanding capsular perforators.

Hou [17] et al. reported no transfusion rate for both Thulium and bipolar enucleation in comparison to robotic simple prostatectomy, which aligns with our result showing a better hemostatic profile of bipolar over thulium enucleation in such a large volume gland, with a statistically significant difference (*p* = 0.0002). Similarly, a systematic review by DeCao [18] and colleagues revealed a significant difference in the blood transfusion rate between Thulium resection of the prostate (TMLRP) and monopolar TURP but not between TMLRP and Bipolar TURP (TUPKP). This could shed light on the literature discrepancy regarding the hemostatic safety profile of bipolar energy, which is superior in our cohort, contradicting other literature enucleation studies. This difference may be attributed to smaller prostate size, the use of monopolar devices, and the non-morcellation resection techniques in these studies.

The early apical release was introduced by Saitta et al. [19] in conjunction with the Enbloc technique to preserve sphincteric mucosa, presuming to lower the rate of post-operative stress incontinence. To our best knowledge, this is the first study addressing Collins knife utilization during early apical release in BipolEP. Using the Collins bipolar knife to perform early apical release, as invented and widespread by HoLEP-based studies, was feasible to lower sphincteric traction during surgery. Our rate of stress incontinence was (8% vs 7%) in the 1st follow-up that dropped down to (3.3% vs. 1.6%) at following follow-up in BipolEP and ThuLEP groups with an insignificant difference. The large prostate volume was presented as one of predictors of postoperative increases incidence of stress incontinence after HoLEP [20]. Utilizing early apical release introduced by many authors [21] through early complete separation of the prostate apex did proof to provide less stress incontinence rates in our study.

In the current study, we found no appreciable difference between the two groups at postoperative follow-up for the IPSS, PVR, and Q max scores, which improved significantly, compared to preoperative values, as expected with any anatomical enucleation of the prostate. Study limitations included the absence of postoperative PSA measurements, the inability to measure hemoglobin concentration in the irrigation fluid, the percentage of patients on 5-alpha-reductase inhibitors, and the limited number of previous studies available for result comparisons.”

## Conclusion

Both BipolEP and ThuLEP, with early apical release, provided a safe and effective management of large-size prostate. However, BipolEP showed less intraoperative irrigation volume and postoperative hemoglobin drop over the ThuLEP group.
